# *KRAS* mutational concordance between primary and metastatic colorectal adenocarcinoma

**DOI:** 10.3892/ol.2014.2411

**Published:** 2014-08-04

**Authors:** PANAGIOTIS PALIOGIANNIS, ANTONIO COSSU, FRANCESCO TANDA, GIUSEPPE PALMIERI, GRAZIA PALOMBA

**Affiliations:** 1 Department of Surgical, Microsurgical and Medical Sciences, University of Sassari, Sassari I-07100, Italy; 2 Institute of Biomolecular Chemistry, Cancer Genetics Unit, National Research Council, Sassari I-07040, Italy

**Keywords:** colorectal cancer, *KRAS* gene, mutation analysis

## Abstract

*KRAS* mutation analysis is commonly performed on tissue samples obtained from primary colorectal cancers (CRCs). The metastatic lesions of CRC are usually considered as qualitatively similar or even identical to the primary tumors. The aim of this study was to evaluate the spectrum and distribution of *KRAS* mutations in a large collection of CRCs, while also evaluating the concordance of primary and metastatic lesions among available paired specimens from the same patients. A total of 729 patients with histologically confirmed advanced CRC at the University Hospital and Local Health Unit (Sassari, Italy) were included. Clinical and pathological features were obtained from medical records and/or pathology reports. Formalin-fixed, paraffin-embedded tissue samples were used for mutation analysis. Genomic DNA was isolated using a standard protocol; the coding sequence and splice junctions of exons 2 and 3 in the *KRAS* gene were screened by direct automated sequencing. Overall, 219 (30%) *KRAS* mutations were found; 208 (30.1%) were identified in the 690 primary tumors and 11 (28.2%) in the 39 metastatic tissue samples. Among the 31 (4.3%) patients who had paired samples of primary CRC and synchronous or asynchronous metastases, 28 (90.3%) showed consistent mutation patterns between the primary tumors and metastatic lesions. In one case, an additive mutation (Q61L) was found in the metastatic tissue, while two other discrepant cases exhibited a different mutation distribution; Q61H in the primitive lesion and G13V in the metastatic lesion in one case, and a mutated primary tumor (Q61L) and wild-type metastasis in another case. The results of this study confirm that a high concordance exists between the results of *KRAS* mutation analysis performed in primitive and metastatic CRCs; independent subclones may be generated in a limited amount of patients.

## Introduction

Colorectal cancer (CRC) is a neoplastic disease with one of the highest incidence rates worldwide with >1,200,000 cases reported in 2008 (1). CRC is the third most common type of neoplastic disease in males and females, following lung and prostate cancer in males, and breast and cervical cancer in females. CRC also presents the fourth most frequent type of cancer-related mortality following lung, stomach and liver cancer (1,2). An evaluation performed by the National Cancer Institute estimated that the total national expenditure for CRC care amounted to 14.1 billion dollars in 2010 in the USA, accounting for ~12% of the global health care costs in the country; further evidence exists indicating that these costs will continue to grow in the years to come (3,4).

These figures, along with a persistent tendency of incidence rates to increase, depict the relevance of the disease and explain the enormous efforts that have been made in last decades in all fields of preventive, clinical, surgical and molecular science in order to confront the problem. Great responses and results have been obtained in the management of non-invasive and early-stage invasive disease, particularly with the improvements in multidisciplinary endoscopic, surgical, radiotherapeutic and medical oncology protocols. Conversely, progress in the setting of advanced and/or metastatic disease have been less consistent, and the relative survival remains relatively low (2,5). The role of tumor genetics in this setting has been extremely important, particularly in the development of novel therapeutic strategies, based on specific molecular targets.

The most relevant results of ‘targeted therapies’ have been obtained through the comprehension of the pathophysiological mechanisms of oncogenesis due to genetic modifications involving the epidermal growth factor receptor (EGFR)-RAS cascade. The EGFR is a transmembrane protein for the epidermal growth factor that explicates its functions through the activation of the RAS protein family (HRAS, KRAS and NRAS). Activated RAS proteins promote cell proliferation through several mechanisms, including constitutive stimulation of the mitogen-activated protein kinases (6). EGFR-targeted agents that compete with EGF for binding to the receptor have been employed in clinical practice, in order to reduce cell proliferation (7). Oncogenic RAS activation is due to specific mutations in the kinase regions of the genes, producing a constitutive induction of the phosphorylating function of the RAS proteins which, in turn, promote neoplastic proliferation and markedly reduce the effect of EGFR-targeted agents (7,8). Mutation of *KRAS* has been demonstrated to act as a predictor of the absence of response to EGFR-targeted agents (9,10). These observations led to a requirement for *KRAS* mutation analysis to be conducted in all patients with CRC, and it is currently performed routinely in clinical practice. However, response rates to EGFR-targeted agents remain low, accounting for <20% of cases (11).

Such a low response rate may be explained on the basis of individual biological behavior or genetic background. In this sense, CRC lesions may arise through acquisition of different molecular alterations; activation of oncogenes which differ from those included in *KRAS*-depending pathways, as well as inactivation of specific tumor suppressor genes (such as *APC, p16, p53* and *DCC*) (12,13) or impairment of mismatch repair genes (including *MLH1*, *MSH2* and, to a lesser extent, *PMS2* and *hMSH6*) in the subset of CRCs associated with microsatellite instability (14). Recently, additional pathways are being exploited toward the assessment of the factors involved in the initiation and progression of CRC (15,16).

Finally, prevalence of such alterations may vary during the disease progression phases and among different types of metastasis, with putative changes in the biological behavior of secondary CRC lesions, including response to targeted treatments (13,17). At present, *KRAS* mutation analysis is performed in nearly all cases via tissue samples obtained from primary CRCs. Metastatic CRC lesions are usually considered as qualitatively similar, or even identical to the primary tumors. The aim of this study was to evaluate the spectrum and distribution of *KRAS* mutations in a large collection of CRC tissues, while also evaluating the concordance of primary and metastatic lesions among the available paired specimens from the same patients.

## Materials and methods

### Samples

A total of 729 patients with histologically confirmed advanced CRC and who regularly participated in the follow-up programs at the University Hospital and Local Health Unit (Sassari, Italy) were included in the study. To avoid any bias, CRC patients were consecutively collected between April 2009 and June 2013. Patients were included regardless of age at diagnosis and disease characteristics. No CRC cases included in this series were associated with clinically relevant colorectal polyposis. Details of the 729 patients are shown in Table I.

A Sardinian origin was ascertained in all cases through genealogical studies; for all patients, the place of birth of their parents and grandparents was assessed in order to assign their geographical origin within the island. Clinical and pathological features for the assessment of the disease stage at diagnosis, as well as the onset age and tumor anatomical location were confirmed by medical records and/or pathology reports.

Formalin-fixed, paraffin-embedded (FFPE) tissue samples of the CRC patients were obtained from the archives of the University Hospital and Local Health Unit. Tissue samples were estimated to contain ≥70% neoplastic cells by light microscopy. All patients were informed about the aims of this study and prior to collection of the tissue samples, written informed consent was obtained. The study was reviewed and approved by the ethical review board of the University of Sassari (Sassari, Italy).

### Mutation analysis

All tumor tissues were collected and processed at the laboratory of the Institute of Biomolecular Chemistry of Sassari (Sassari, Italy); genomic DNA was isolated from tissue sections using a standard protocol and DNA quality was assessed for each specimen. In particular, paraffin was removed from the FFPE samples by treatment with Bio-Clear (Bio-Optica, Milan, Italy) and DNA was purified using the QIAamp DNA FFPE tissue kit (Qiagen, Valencia, CA, USA).

The coding sequence and splice junctions of exons 2 and 3 in the *KRAS* gene (where all pathogenetic mutations occur), were screened for mutations by direct automated sequencing. Briefly, polymerase chain reaction (PCR) was performed on 25–50 ng of isolated genomic DNA in a 9,700 Thermal cycler (Applied Biosystems, Foster City, CA, USA); all PCR-amplified products were directly sequenced using an automated fluorescence-based cycle sequencer (ABIPRISM 3100; Applied Biosystems), as described in our previous study (18).

The primer sequences used were as follows: Forward, TGT GTG ACA TGT TCT AAT ATA GTC ACAT and reverse, GGT CCT GCA CCA GTA ATA TGC for *KRAS* exon 2; and forward, GAC TGT GTT TCT CCC TTCT and reverse, TGG CAA ATA CAC AAA GAA AG for *KRAS* exon 3. Protocols for the PCR-based assays were designed and optimized at the laboratory of the Institute of Biomolecular Chemistry at the National Research Council (Sassari, Italy), and are available upon request. A total amount of 25–50 ng of isolated genomic DNA was used for PCR assay, with 0.5 μM of forward and reverse primers for each KRAS codon (2 and 3), 1.5 μM MgCl_2_, 0.2 μM dNTPs and 1 unit of recombinant Taq DNA polymerase (GE Healthcare, Piscataway, NJ, USA). The basic PCR conditions were as follows: 38 cycles of denaturation at 94°C for 30 sec, primer annealing at 55–64°C (depending on the primer) for 30 sec, and polymerase extension at 72°C for 1 min. All PCR reactions were terminated with a 7-minute extension at 72°C.

### Statistical analysis

Statistical analysis was performed using the statistical package SPSS version 7.5 for Windows (SPSS, Inc., Chicago, IL, USA) by Pearson’s χ*^2^* test. The exact coefficient for sample proportion analysis was performed to determine all significant parameters. P<0.05 was considered to indicate a statistically significant different.

## Results

### Distribution of KRAS mutations

In total, 219 (30%) *KRAS* mutations were identified among the 729 analyzed cases. The distribution of the mutations in relation to *KRAS* codons was as follows: 158 (72.1%) in codon 12, 44 (20.1%) in codon 13, 15 (6.9%) in codon 61, and two (0.9%) in other codons.

For patient classification, *KRAS* mutations were detected in 208 of 690 (30.1%) primary tumors and 11 (28.2%) of 39 metastatic tissue samples. The site of metastasis was the liver in 17 (43.6%) cases, the lung in seven (17.9%) cases, the peritoneum or omentum in six (15.4%) cases, the regional lymph nodes in four (10.2%) cases, and the pancreas and vagina in one case (2.6%) each. In the remaining three (7.7%) cases neoplastic tissue was obtained from pelvic recurrences.

### Mutation patterns in paired samples

Among the 31 (4.3%) patients who had paired samples of primary CRC and synchronous or asynchronous metastases, 28 (90.3%) showed consistent mutation patterns between the primary tumors and metastatic lesions. Of the paired samples, the metastatic samples predominantly constituted visceral (liver and lung) lesions, while the three secondary lesions were pelvic recurrences. In nine (29.0%) of the paired cases, *KRAS* mutations were detected; as depicted in Table II. Among the three (9.7%) paired cases with discrepancies in the *KRAS* mutation pattern between primary and secondary tumors, one presented an additive mutation (Q61L) in the metastatic tissue, one carried different mutation types (Q61H in the primitive lesion and G13V in the metastatic one), and one exhibited a mutated (Q61L) primary tumor and wild-type metastasis (Table II). Among the remaining 708 unpaired patients, one case (0.1%) only presented coexistence of two *KRAS* mutations (G12D and Q61L) in the same tumor tissue.

## Discussion

Targeted therapies have been recently introduced in medical oncology for the treatment of several malignant neoplasias. The most diffused currently in use are tyrosin kinase inhibitors for EGFR-mutated lung adenocarcinomas, trastuzumab for HER2-negative breast cancer and cetuximab or panituximab for *KRAS*-mutated CRC (19). The use of targeted agents imposed the molecular testing of genes confirmed to be involved in the pathogenesis of tumors.

Molecular testing for the assessment of targeted treatment is generally performed on tissue obtained from the primary lesion. This is particularly true for CRC, as endoscopic methods are widely used and offer the possibility to obtain consistent neoplastic tissue samples. Molecular analysis on the primary tumor in metastatic patients was considered effective in the past, as metastases were supposed to maintain the biological features of the primary lesions. This concept was based on previous evidence that primary and metastatic lesions share numerous morphological and immunohistochemical features, allowing pathologists to obtain a diagnosis, and that proliferation rates were generally similar in primitive and secondary neoplastic lesions (20). More recent acquisitions guide to a new pathophysiological vision of tumorigenesis, characterized as a dynamic process that continuously changes and evolves during the progression of the disease (21).

These acquisitions are principally based on several differences identified within the primary mass itself, and within the primary tumor and its subsequent distant metastases. Morphological and immunohistochemical variability in the context of a primary tumor is very common. Different areas of proliferation, cell cycle arrest, epithelial differentiation, cell adhesion and dissemination are often detected in a single tumor (22). These differences may influence the outcomes of molecular analyses, considering that such analyses are generally performed on a limited fraction of tumor tissues. Studies confronting molecular analyses on different samples of the same tumor tissues are required to establish the manner in which intratumoral heterogeneity impacts the results of molecular tests. In the present series, which included 729 patients with advanced CRC, only two cases with intratumoral variability of the mutation status (coexistence of mutations in *KRAS* exons 2 and 3; G12D + Q61L and G13V +Q61L) were identified, accounting for the 0.3% of all examined cases.

Discrepancies between the primary and secondary neoplastic lesions have been reported for several types of tumors. HER 2, estrogen receptor and progesterone receptor (PR) expression in patients with advanced stage breast cancer has been investigated by Vignot *et al* (23); marked discrepancies were found for PR expression, however, no definitive conclusions could be drawn, particularly with regard to the clinical impact of such findings. Shah *et al* (24) studied the metastatic mutational evolution in a single case of lobular breast cancer, evidencing 19 novel non-synchronous coding mutations of the 32 found in the metastasis, while Ding *et al* (25) identified two *de novo* mutations in the metastasis of a patient affected with a basal-like carcinoma of the breast. With regard to the non-small cell lung cancer, the discordance rates of *EGFR* mutations vary between 10 and 54%; however, these figures must be interpreted with caution, given the heterogeneity of methods employed in various studies (23). Two studies have investigated the genomes of primary and metastatic pancreatic tumors, evidencing clonal expansions in the metastatic tumors and suggesting that mutations in cancer-driving genes may occur later in oncogenesis, and may alter the biological behavior of the lesions (26,27).

Concerning CRC, several studies have investigated genetic heterogeneity between primary and secondary lesions. Goranova *et al* (28) demonstrated variations in APC, KRAS and TP53 expression, suggesting a certain heterogeneity, as was evidenced in this study; one patient presented a discordant *KRAS* mutation, while another carried an additional mutation. However, it is important to comprehend whether, as well as the manner in which this heterogeneity impacts on clinical practice. Neumann *et al* (29) examined *KRAS* mutational status in 879 primary CRC specimens and 139 metastasis specimens, but did not identify any significant discrepancies. Knijin *et al* (30), Cejas *et al* (31) and Santini *et al* (32) in their series of 305, 110 and 99 cases of paired primary and metastatic lesions, respectively, demonstrated consistency rates of ≤96.4% (30–32). Similar results were also found in numerous smaller series (Table III), suggesting that molecular heterogeneity in primary and secondary CRC does not impact on the current clinical practice, oriented to the identification of candidates for targeted therapies (33–40). A meta-analysis including a total of 19 publications with ~1,100 paired primary and secondary CRC tissues has recently confirmed an extremely high concordance of the *KRAS* genotype in primary and metastatic tumors, while also showing the highest rates of discrepancies in lymph node metastases as compared with correspondent primary CRC (41). Overall, either type of tumor tissue, primary or secondary CRC (with a putative exception for metastatic lymph nodes), could be useful as a source to detect *KRAS* mutations in selecting patients to be addressed to anti-EGFR therapy.

The proportion of the discordant cases in the majority of reports published ranges between 0 and 12%; three (9.7%) paired samples out of 31 analyzed patients demonstrated discrepancies in *KRAS* mutation patterns between primary and secondary tumors in this study. In one discrepant case, a wild-type primary tumor and mutated metastasis, suggested that mutations in such a gene may be acquired during disease dissemination. In the other two discrepant cases, wild-type metastasis was observed in one patient with mutated primary tumors and a different mutation pattern was observed in one patient between the melanoma lesions (Q61H in the primary and G13V in secondary tumor). Altogether, it could be speculated that discrepant metastases may depend on the molecular heterogeneity of the cell types in a limited subset of primary CRCs.

Although the current practice in the detection of *KRAS* mutations can be considered accurate, some aspects must be addressed in the future. The most relevant is to establish clear protocols in mutational analysis methods in order to avoid technical imprecisions and to accurately identify mutations that may play a key role in the metastatic process in small tissue samples of primary tumors. Employment of novel methods and technologies for molecular analysis will ameliorate the current knowledge on the clinical significance and utility of genetic modifications and discrepancies arising in the course of neoplastic diseases (23,42). This is likely to improve clinical practice allowing the identification of the best treatment strategy for all patients.

In conclusion, the results of the current study suggest that a high concordance exists between the results of *KRAS* mutation analysis performed in primitive and metastatic or recurrent neoplastic tissues in primitive and metastatic, or recurrent neoplastic tissues of patients with advanced CRC. A small proportion of non-concordant cases may occur and may be attributed to additional molecular events that arise as the metastatic process advances. The techniques currently in use for mutation analysis offer globally high-quality results, contributing substantially in the correct application of targeted therapies in clinical practice.

## Figures and Tables

**Table I:** Characteristics of the 729 colorectal cancer patients at diagnosis.

Characteristic	n (%)
Gender	
Male	443 (61)
Female	286 (39)
Tumor site	
Right-transverse colon	261 (36)
Left colon	294 (40)
Rectum	174 (24)
Disease stage	
II	259 (36)
III	248 (34)
IV	222 (30)
Tumor grading	
Well-differentiated	86 (12)
Moderately differentiated	583 (80)
Poorly differentiated	60 (8)
Age, years	
<50	83 (11)
50–69	438 (60)
≥70	208 (29)

**Table II:** Types of *KRAS* mutation among patients in whom molecular analysis was performed in the primary and metastatic lesions.

Patient no.	Primary tumor	Metastasis
1	G13V	G13V + Q61L
2	G13D	G13D
3	G13D	G13D
4	Q61H	G13V
5	Q61L	Wild-type
6	G12V	G12V
7	G12D	G12D
8	G13D	G13D
9	G12D	G12D

**Table III:** *KRAS* mutation consistency between primary and metastatic lesions as depicted in the current literature.

First author (ref.)	Year	Paired cases examined, n	Consistency, %
Knijn (30)	2011	305	96
Cejas (31)	2009	110	94
Santini (32)	2008	99	96
Etienne-Grimaldi (33)	2008	48	100
Park (34)	2011	17	76.5
Watanabe (35)	2011	43	88
Molinari (36)	2009	37	92
Loupakis (37)	2009	43	93
Mariani (38)	2010	38	97
Baldus (39)	2010	20	90
Italiano (40)	2010	59	95
